# Breast Primary Follicular Lymphoma: Two Cases of a Rare Entity Treated With Radical Radiotherapy and Rituximab

**DOI:** 10.7759/cureus.95870

**Published:** 2025-11-01

**Authors:** Luísa Samarão, José Mário Mariz, Joana Gonçalves, Ângelo Martins, Carla Castro

**Affiliations:** 1 Radiation Oncology, Portuguese Oncology Institute of Porto, Porto, PRT; 2 Hematology and Bone Marrow Transplantation, Portuguese Oncology Institute of Porto, Porto, PRT

**Keywords:** breast, management, primary follicular lymphoma, radiotherapy, rituximab

## Abstract

Primary breast lymphoma (PBL) is a rare form of non-Hodgkin's lymphoma (NHL), characterized by lymphoma primarily affecting the breast tissue and not seen anywhere else in the body. Follicular breast lymphoma, a distinct histological variant of NHL, manifests with an even greater rarity within the context of breast tissue. The optimal therapeutic approach for these tumors remains controversial. Two cases of follicular lymphoma of the breast are hereby reported. The patients, aged 58 and 56 years, presented at the institution with a breast lump. A biopsy was subsequently performed, yielding a diagnosis of follicular lymphoma. The subjects underwent a positron emission tomography (PET)-computed tomography (CT) scan, which revealed hypermetabolic uptake in the respective breast nodules, with no further alterations. Consequently, they were staged as stage IE. Patients were offered a course of radical radiotherapy with concomitant rituximab. PET-CT scans five months after treatment from both patients revealed a complete metabolic response. In conclusion, we emphasize the role of radiotherapy and rituximab in the management of follicular breast lymphoma.

## Introduction

Lymphoma of the breast can be classified into two categories: primary, in which the breast is the primary site of involvement, and secondary to systemic lymphoma [1]. Primary breast lymphoma (PBL) is a rare form of cancer with an estimated incidence of 1-2% of all non-Hodgkin lymphomas and between 0.04% and 0.5% of all breast cancer cases [2,3]. Histologically, the most common subtype of PBL is diffuse large B-cell lymphoma (DLBCL), followed by follicular and mucosa-associated lymphoid tissue (MALT) lymphoma [4].

The most common age range for PBL diagnosis is between 60 and 65 years [5]. A painless breast mass, most commonly located in the outer quadrant of the breast, is the main clinical manifestation of PBL. However, in 12% of cases, the patients are asymptomatic, and the diagnosis is made incidentally on a mammogram [6]. It is difficult to distinguish clinically or radiologically from other primary breast cancers, and diagnosis relies mainly on histologic evaluation after core biopsy.

Regarding treatment, there is no consensus on the ideal therapeutic modality: it may be based on a combination of radiotherapy (RT), chemotherapy, and immunotherapy, depending on the histologic subtype and stage [7].

In this case report, we describe two cases of a 56-year-old woman and a 58-year-old woman with primary follicular lymphoma of the breast treated at our institution with radical RT and rituximab with complete clinical response.

These two cases were previously presented as a meeting abstract at the Perspectives in Oncology Meeting, held in Portugal from the 15th to the 17th of February, 2024.

## Case presentation

Case 1

A 56-year-old woman was referred to our institution due to the diagnosis of a breast lump on a screening mammogram. On physical examination, a 2 cm palpable nodule was identified in the upper inner quadrant of the right breast, with no evidence of axillary adenopathy or other significant alterations. With regard to medical and surgical history, she had diagnoses of obesity and chronic gastritis and had previously undergone a cholecystectomy. She underwent menopause at 53 years old. She is on esomeprazole for chronic gastritis.

The patient underwent a biopsy of the breast lump. Histopathological examination (HPE) revealed a diagnosis of grade 2 nodular follicular lymphoma.

**Figure 1:**
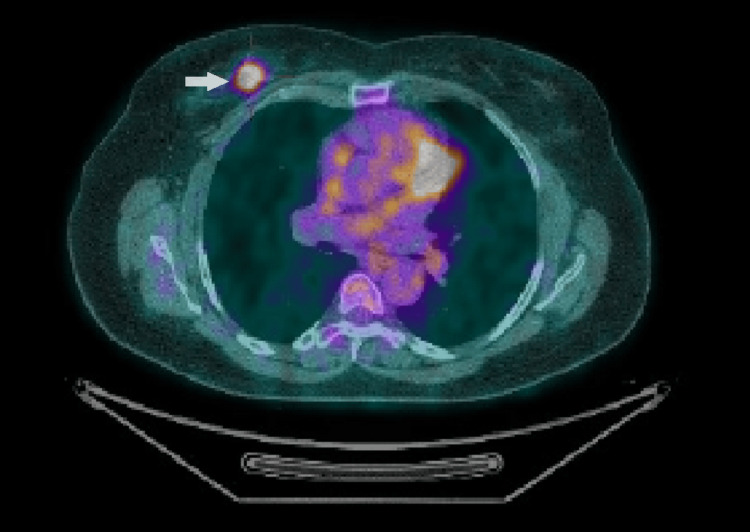
18F-FDG PET-CT before treatment revealing hypermetabolic activity in the upper inner quadrant of the breast 18F-FDG PET-CT: 18F-fluorodeoxyglucose positron emission tomography-computed tomography

Subsequently, the patient underwent an 18F-fluorodeoxyglucose positron emission tomography-computed tomography (18F-FDG PET-CT), which revealed a soft tissue density nodule with hypermetabolic activity (maximum standardized uptake value (SUVmax): 6.3) (Figure 1) in the upper inner quadrant of the right breast. There was no other abnormal hypermetabolic focus in the regions of the body scanned. In view of the HPE report, the lesion in the right breast corresponds to lymphoproliferative disorder: PBL.

A CT study of the chest, abdomen, and pelvis demonstrated a density in the inner quadrant of the right breast measuring 23×19 mm. The bone marrow biopsy showed no evidence of malignancy. The patient was staged as IE, according to the Ann Arbor staging system.

A multidisciplinary team proposed treatment with curative-intent RT with associated rituximab. The delivered dose was 24 Gy in 12 fractions, to the whole breast (right), with a simultaneous integrated boost (SIB) to the tumor of 29 Gy, in the same 12 fractions, at 2.41 Gy/day, using 6 MV photons, with an intensity-modulated radiation therapy (IMRT)-SIB dosimetric plan. She was treated for 16 days, with no interruptions, experiencing only grade 1 radiation dermatitis throughout the treatment sessions. The patient initiated rituximab treatment concomitantly, resulting in a total of four cycles administered weekly.

**Figure 2:**
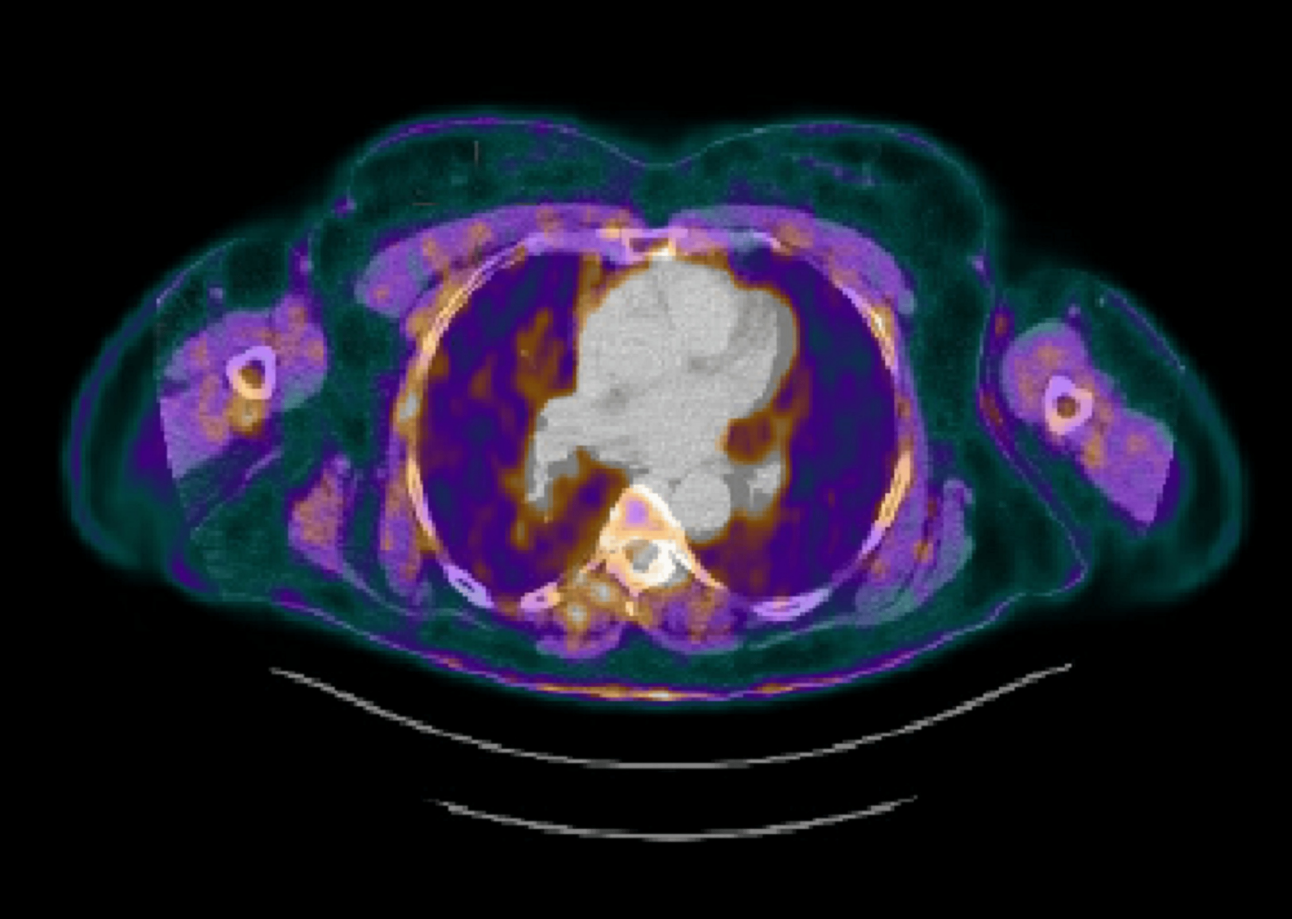
18F-FDG PET-CT after treatment, where a complete response is visible 18F-FDG PET-CT: 18F-fluorodeoxyglucose positron emission tomography-computed tomography

Post-treatment PET-CT (five months after RT) revealed a complete metabolic response (Figure 2).

Case 2

A 58-year-old woman was referred to our institution after detecting a palpable painless lump in her right breast. Mammography and ultrasound suggested two suspicious nodules, with a diameter of 15 mm and 7 mm, respectively, in the transition of the outer quadrants (TOQ) of the right breast (Breast Imaging-Reporting and Data System (BI-RADS) 4). On physical examination, two palpable nodules were identified in the TOQ, with one measuring approximately 2 cm and the other measuring less than 1 cm. No additional abnormalities were observed during the physical examination. With regard to medical and surgical history, she was a current smoker, had depression and dyslipidemia, and had previously undergone an appendectomy. She underwent menopause at 47 years old. She was taking the following medication: alprazolam, escitalopram, omeprazole, mirtazapine, and pitavastatin.

**Figure 3:**
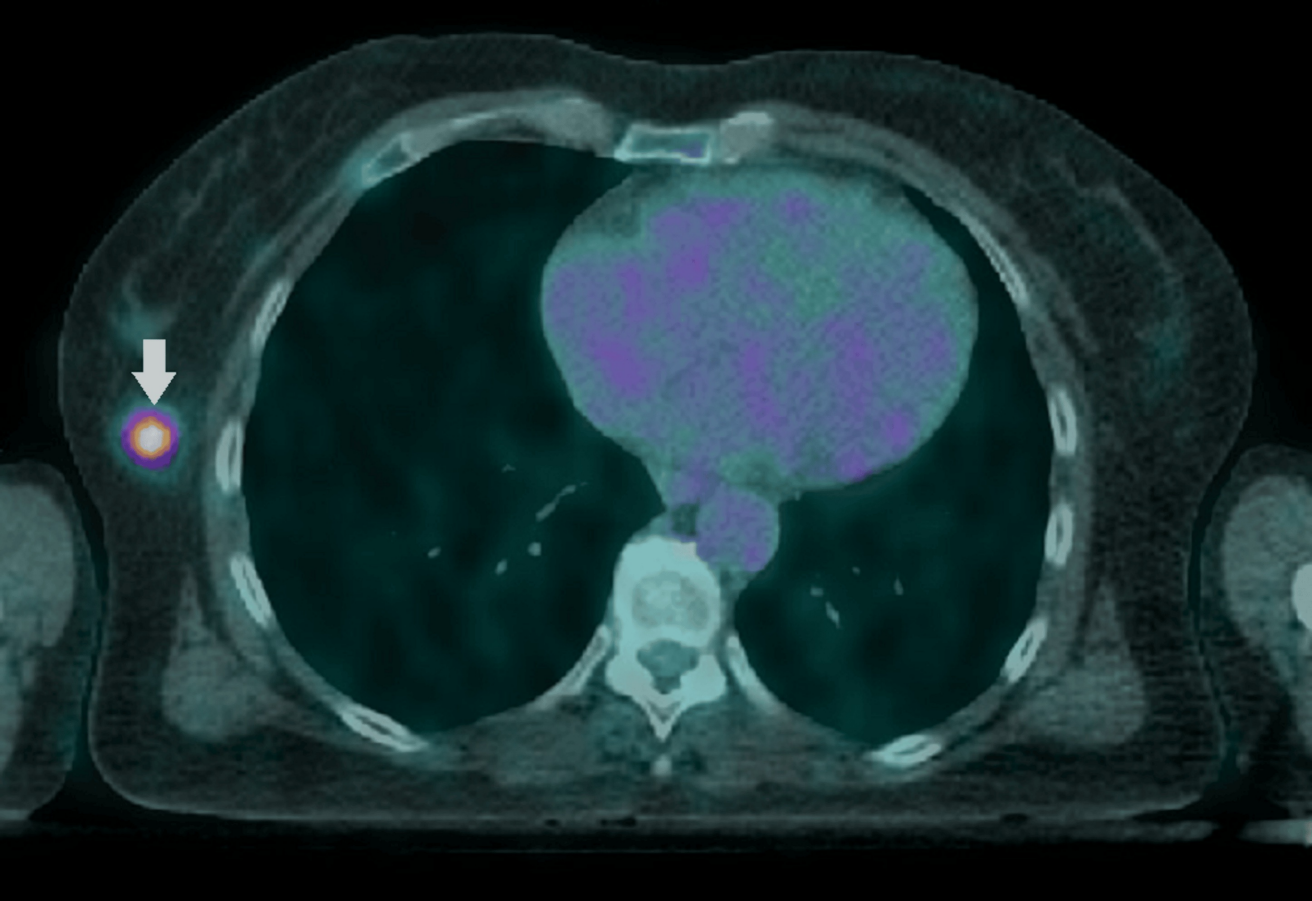
Staging 18F-FDG PET-CT showing hypermetabolic activity at the transition of the outer quadrant of the right breast 18F-FDG PET-CT: 18F-fluorodeoxyglucose positron emission tomography-computed tomography

The core biopsy of the right breast nodules (15 mm) indicated a diagnosis of grade 1 nodular follicular lymphoma. The PET-CT demonstrated increased metabolic activity of 18F-FDG (SUVmax: 5.70) in two nodules located at the periphery of the TOQ of the right breast (Figure 3).

The patient was consequently staged as IE (Ann Arbor staging) and discussed in a multidisciplinary group, where RT and rituximab were agreed upon. The dose and fractionation of RT were the same as in the case described above. The patient was treated for a period of 16 days, with no interruptions and without complications.

**Figure 4:**
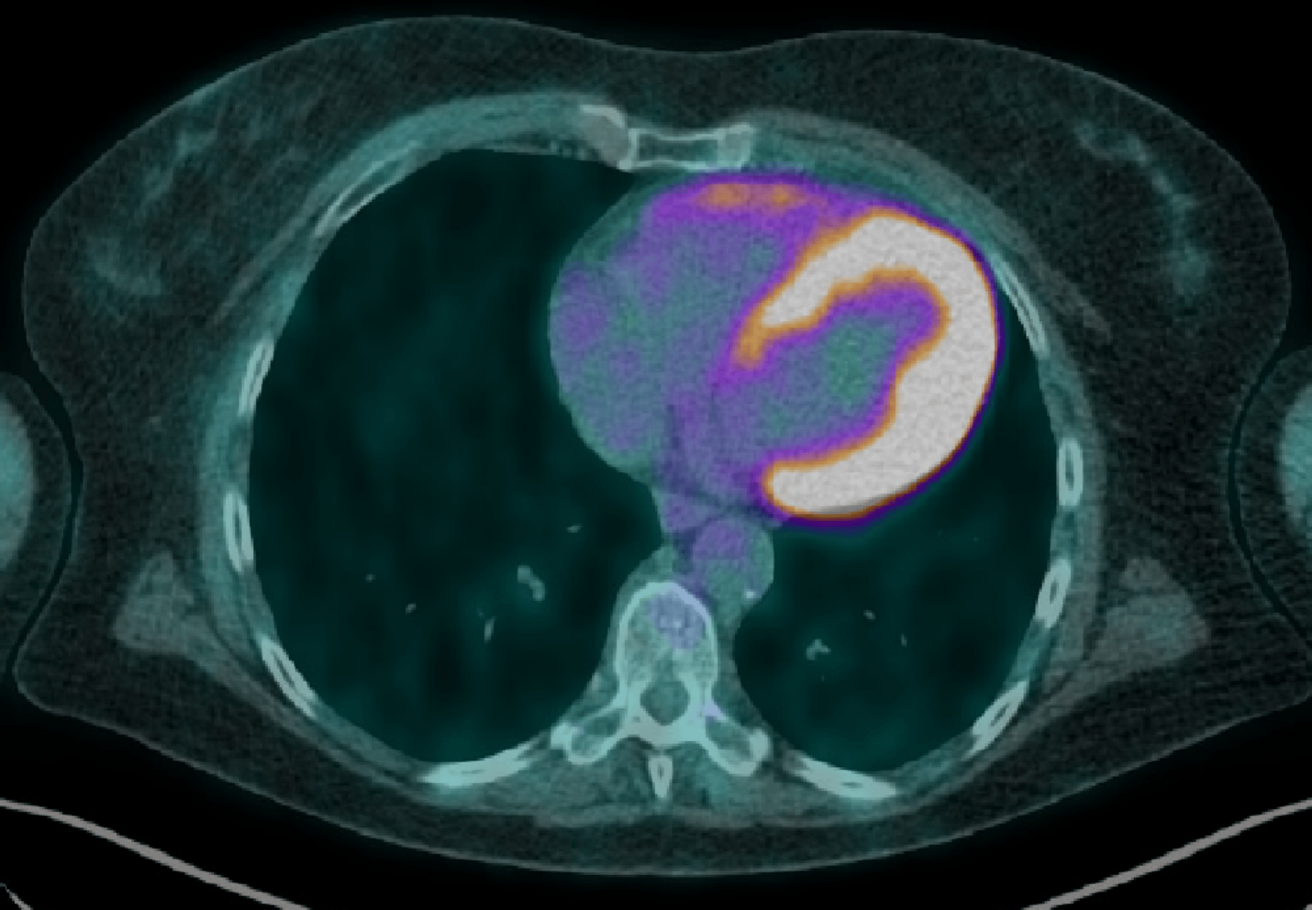
18F-FDG PET-CT after treatment with no hypermetabolic activity, resulting in a complete response 18F-FDG PET-CT: 18F-fluorodeoxyglucose positron emission tomography-computed tomography

A post-treatment PET-CT, performed six months after the completion of RT, revealed a complete metabolic response (Figure 4).

The patients are regularly followed to monitor disease status and treatment-related adverse events. Two years later, they are alive and being monitored clinically and with mammograms, with continued complete clinical responses without evidence of recurrence.

The presented cases are noteworthy because they involve a primary lymphoma of the breast with a rare histology treated with radical RT and rituximab with a favorable outcome.

## Discussion

PBL is a rare tumor, comprising approximately 1% of all non-Hodgkin lymphomas and 2% of extranodal lymphomas [8]. DLBCL is the most prevalent histology of PBL (40-70%) [4]. Follicular and marginal zone breast lymphomas are less prevalent, accounting for 14% and 9% of patients with PBL, respectively [9].

Wiseman and Liao, in 1972, were the first to define PBL, establishing the following diagnostic criteria: the principal site of presentation was the breast; there was an absence of prior lymphoma history or current evidence of metastatic disease at the time of diagnosis; a confirmed diagnosis of lymphoma within the affected breast tissue was made; and ipsilateral lymph node may be involved at the same time of diagnosis of PBL [10].

PBL is mainly characterized by the presence of a painless breast mass, with or without ipsilateral axillary lymph node involvement [11]. The clinical presentation is generally analogous to that of primary breast cancer [12]. The disease predominantly (95-100%) affects women in the fifth to sixth decades of life [13]. In our cases, consistent with the existing literature, we observed two women in their sixth decade of life with a diagnosis of PBL. One patient presented with a painless breast lump, while the other patient was asymptomatic and was diagnosed during a screening procedure.

Regarding imaging, mammography and ultrasonography of PBL are non-specific and bear a resemblance to those observed in cases of breast cancer. PET-CT scans are crucial for tumor staging and the assessment of treatment response [14]. In the present cases, both patients underwent PET-CT scans for staging and the evaluation of treatment response, which revealed a complete metabolic response.

The treatment of PBL is not yet well established, and it depends on the histological subtype and stage. Regarding surgery, a mastectomy for PBL is not recommended because there are no survival benefits. The procedure is only offered in certain cases for diagnostic purposes, and minimally invasive surgery is the preferred option [15]. In a retrospective analysis of 47 women with PBL, Luo et al. demonstrated that complete resection of the primary lesion did not result in a significantly improved prognosis. The explanation proffered by the authors is that lymphoma is a hematologic malignancy and its pathogenesis and progression will differ from that of solid tumors, benefiting more from systemic treatment than surgery [16].

RT has an important role in the treatment of PBL. A multicentric retrospective study from the International Extranodal Lymphoma Study Group included 60 cases of PBL: 36 follicular and 24 marginal zone lymphomas. In this study, surgery, chemotherapy, and RT, alone or in combination, were used as first-line treatments in 67%, 42%, and 52% of patients, respectively. The RT dose range used was 25-50 Gy (median: 38 Gy), with nine patients with local disease treated with RT alone as a first-line treatment showing no recurrence in irradiated fields [17]. Ganjoo et al. presented a retrospective study of 37 patients with PBL, including seven cases of follicular lymphoma. Of the cases examined, only one patient with follicular lymphoma was staged as IE, analogous to the case presented. The patient was treated with localized RT alone, receiving 36-50.40 Gy, including the breast and axilla and after 5.8 years exhibited no signs of recurrence. The remaining six cases of follicular lymphoma with IIIE and IV were treated with a combination of radiation and either CHOP (cyclophosphamide, doxorubicin, vincristine, and prednisone)/CVP (cyclophosphamide, vincristine, and prednisone) or rituximab [18]. A study cohort from the Surveillance, Epidemiology, and End Results (SEER) database included a total of 386 patients with stage I and II PBL-DLBCL to determine if consolidative RT could provide additional therapeutic benefits for patients treated with rituximab. The authors concluded that adding RT to rituximab was associated with improved overall survival [19]. However, our cases are follicular breast lymphomas, and due to the rarity of this disease, there are no prospective trials, as far as we know, to compare the efficacy of rituximab with RT in this setting. Therefore, we must extrapolate from retrospective trials that include different histologies.

## Conclusions

Although breast primary follicular lymphoma is a rare entity, it must be considered in the differential diagnosis of breast tumors. It is essential to emphasize the critical role of histopathological and immunohistochemical confirmation in the diagnostic workup of breast masses that are suggestive of lymphoma. The cases presented allow for reflection on the different therapeutic modalities in PBL, mainly follicular histology, highlighting the role of radical RT and rituximab in the treatment of these tumors. Further studies are necessary to elucidate the efficacy of radical RT in this context.
